# New generation of bioreactors that advance extracellular matrix modelling and tissue engineering

**DOI:** 10.1007/s10529-018-2611-7

**Published:** 2018-10-27

**Authors:** Shehnaz Ahmed, Veeren M. Chauhan, Amir M. Ghaemmaghami, Jonathan W. Aylott

**Affiliations:** 1 School of Pharmacy, University of Nottingham, Boots Sciences Building, University Park, Nottingham, UK; 2 School of Life Sciences, University of Nottingham, Life Sciences Building, University Park, Nottingham, NG7 2RD UK

**Keywords:** Bio-sensing, Bioreactor, Extracellular matrix, Nanosensors, Regenerative medicine, Scaffolds, Tissue engineering

## Abstract

Bioreactors hold a lot of promise for tissue engineering and regenerative medicine applications. They have multiple uses including cell cultivation for therapeutic production and for in vitro organ modelling to provide a more physiologically relevant environment for cultures compared to conventional static conditions. Bioreactors are often used in combination with scaffolds as the nutrient flow can enhance oxygen and diffusion throughout the 3D constructs to prevent the formation of necrotic cores. A variety of scaffolds have been fabricated to achieve a structural architecture that mimic native extracellular matrix. Future developments of in vitro models will incorporate the ability to non-invasively monitor the cellular microenvironment to enhance the understanding of in vitro conditions. This review details current advancements in bioreactor and scaffold systems and provides insight on how in vitro models can be augmented for future biomedical applications.

## Introduction

Tissue engineering is the science of repairing, replacing and enhancing functional properties of biological tissue, such as diseased or damaged organs, through the combination of cells, biologically active molecules, synthetic and innate biological components (The National Institute of Biomedical Imaging and Bioengineering 2016; Katari et al. 2015; Okamoto and John 2013).

Tissue engineered constructs can act as model systems that permit investigation and mimic specific cellular processes and interactions to further improve our understanding and subsequently develop future therapeutics. The development of in vitro tissue models allows predictions on drug activity, metabolism and toxicity in vivo to be made which is important for drug discovery (Maltman and Przyborski 2010). The pharmaceutical industry in particular is in need of more physiologically relevant and accurate models due to the rising cost-to-delivery ratios and poor predictive value of existing in vitro tests (Maltman and Przyborski 2010).

When engineering a tissue, recreating and controlling the overall cellular microenvironment is essential as this can strongly influence cell behaviour (Ozcelik et al. 2014). The cellular microenvironment is made up by factors that directly affect conditions around a cell or a group of cells, which have direct or indirect effect on cell behaviour via biophysical, biochemical or alternate pathways (Ozcelik et al. 2014). There are three main types of cues within the cellular microenvironment including biochemical, physiochemical and mechano-structural as given in Table 1 (Sbrana and Ahluwalia 2012). These can be controlled by using in vitro design and engineering. Tissue constructs are developed in a variety of forms utilising different types of substrates, cell types and culture conditions to suit a range of specific applications. Birnbaum suggests a variety of components when combined together can create a more biologically relevant 3D tissue model compared to conventional 2D culture models as given Table 2 (Birnbaum 2011). However, due to technical challenges and complex interplay between the components it can be difficult to produce functional and mature tissue models incorporating all features. The main components required for tissue engineering include cells/tissues, scaffolds, bioreactors and the ability to monitor the cellular environment. When choosing a bioreactor for cell culture it is important to consider the scale of manufacture based on whether the utility of the bioreactor is for research or clinical purposes, which may require small or large-scale batch sizes, respectively. Small-scale culture of cells is typically used for in vitro research studies based on micro and milli scale volumes. Large scale culture for clinical use have been developed with volumes of up to 20,000 litres (Harrison and Chauhan 2018). Some examples of large scale production for clinical applications include adipose-derived stromal cells for tissue engineering (Haack-Sørensen et al. 2018), human induced pluripotent stem cells for drug screening regenerative medicine (Yamashita et al. 2018), megakaryocytic progenitor cell line for regenerative medicine (Retno Wahyu et al. 2018) and mesenchymal stem cell for cartilage tissue generation (Daly et al. 2018).

**Table 1:** The biochemical, physiochemical and mechano-structural cues along with their factors present in the cellular microenvironment

Cue	Factors
Biochemical	Cytokines
	Other cells
	Hormones
	Nutrients
	pH
Physio-chemical	Oxygen
	Temperature
	Surface energy
	Flow
Mechano-structural	Shear stress
	Strain
	Stiffness
	Roughness
	Topography
	Architecture

**Table 2:** Generalised components required to make a 3-Dimensional model to mimic in vivo biological systems

Component	Details
Scaffold	Purified ECM, synthetic polymers, composites
Cells	Stem/progenitor, differentiated, mixed cell types
Structure	Porosity, topography, stiffness
Spatial/temporal patterning	Cytokines gradients, controlled release
Perfusion	Embedded channels, vascularisation
Bioreactors	Optimised culture conditions, biomechanics
Innervation	Signal propagation, coordinated response
Host response	Generalised inflammation, specific immunity
Functional readout	Real time, label free, non-destructive sensing, imaging
Computational framework	Systems integration, multi-scale modelling, simulation, feedback

## The extracellular matrix (ECM)

**Fig. 1:**
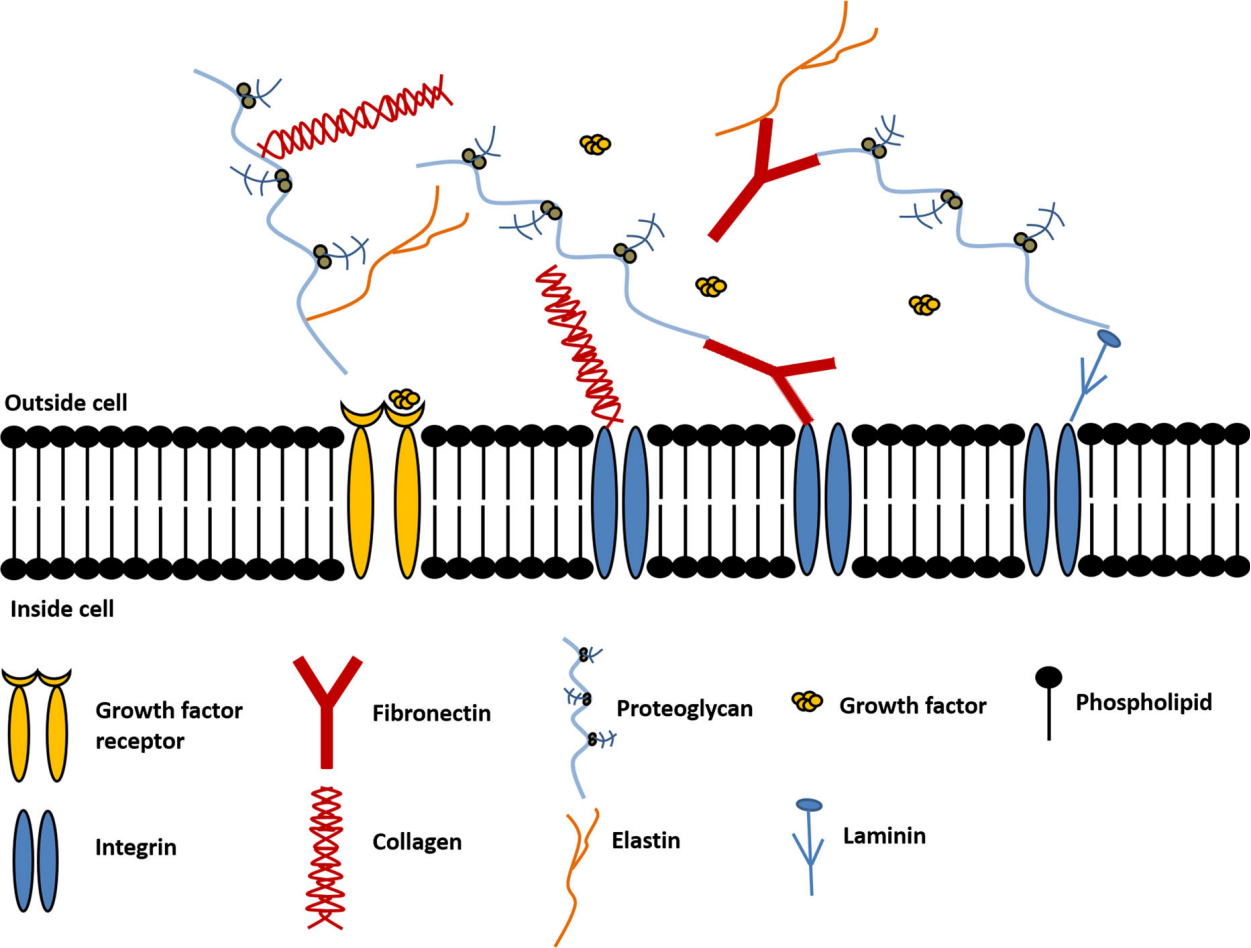
Extracellular matrix extending outside the cell membrane, components include collagen, fibronectin, elastic, laminin and proteoglycans

A major component of tissue volume is the extracellular space, which contains a network of extracellular matrix (ECM) proteins and polysaccharides that surround the cells (Lberts et al. 2002). A schematic showing the main constituents of the ECM are displayed in Fig. 1. These include collagens, elastin, fibronectin, laminins, glycoproteins, proteoglycans and glycosaminoglycans, which are produced intracellularly by resident cells and secreted by exocytosis (Theocharis et al. 2016; Lewin et al. 2007).

Naturally occurring ECM provides inductive signals that may guide cell phenotype determination and/or cell adhesion, proliferation, and survival (Neal et al. 2018). The requirement of cells to attach to the ECM for cell growth is referred to as anchorage dependence, which is mediated mainly by integrins and the intracellular signals they generate. Integrins are transmembrane receptors that facilitate interactions between the ECM and the actin cytoskeleton during cell motility and adhesion. Binding specificity is regulated by the extracellular domain of integrins that recognise ligands such as Arg-Glyc-Asp motif (RGD) found on fibronectin (Chan et al. 2007). Electrospun scaffolds have been used in tissue engineering to mimic the structural framework of the extracellular matrix to act as a template for cell growth. Their fibrous porous structure can facilitate cell growth and proliferation, creating more physiologically relevant 3-Dimensional (3D) in vitro models, compared to conventional 2D culture in well plates.

## Tissue engineering

Tissue engineered systems can be described as three-dimensional porous solid biomaterials (Dhandayuthapani et al. 2011) where cells can be seeded and the scaffold construct will act as template for tissue regeneration to guide the growth of new tissue (Plunkett and O’Brien 2011). Decellularised extracellular matrices are often referred to as the gold standard of scaffolds. These are biological scaffolds derived from native whole tissues that have had the cellular components removed leaving behind micro and macro-scale structural components and functional ECM proteins. This provides the cues necessary for cellular processes such as adhesion, proliferation and maturation. However, challenges come with using decellularised matrices including the sterilisation process which should avoid damaging the ECM ultrastructure and mechanical properties. In addition, ECM scaffolds alone, and their degradation can induce a host innate immune response (Taylor et al. 2018).

Scaffolds can be fabricated by a variety of methods including electrospinning and 3D printing techniques to create the morphology and dimensions to mimic the native extracellular matrix (Harrington et al. 2014). They also can be composed of natural polymers, synthetic material or natural-synthetic material hybrids. Natural polymers include purified extracellular matrix proteins, which provide biological cues for cell attachment and activity, whilst synthetic polymers give the mechanical support required to maintain the structural framework of the scaffold. When designing the scaffold, parameters such as porosity, topography and stiffness should also be considered as these can affect cell behaviour, e.g. cellular differentiation (Ghasemi-Mobarakeh 2015).

Scaffolds provide the necessary support for cells to maintain viability, proliferate, and differentiate into specific cells, and determine the morphology of the resultant tissue. The attachment, proliferation and differentiation of cells are strongly affected by the microenvironment associated with a scaffold, including the size, geometry, density of the pores, the “windows” connecting the pores and the surface properties (Choi et al. 2010). Scaffolds can act as a template of the extracellular matrix (ECM) to guide cell attachment and tissue formation thus providing a platform for structural support (Plunkett and O’Brien 2011).

The complexity of the network of ECM proteins emphasises the importance of preparing a platform that can mimic the structural features of the ECM to facilitate cellular processes including cell adhesion, proliferation and differentiation (Wang et al. 2013). Scaffolds should be porous, to allow efficient nutrient and oxygen diffusion to achieve high cell viability without compromising the mechanical integrity of the scaffold (Chan and Leong 2008). If the scaffold is being used for implantation then the scaffold should not induce a severe inflammatory response, as this could reduce healing or cause rejection in the body. Furthermore, scaffolds for implantation should be biodegradable, as the aim is to support the body’s own cells to produce their own ECM and replace the implanted tissue engineered construct. Moreover, the by-products of this degradation should be biocompatible, so that it is nontoxic to the body, and biodegradable.

The chosen scaffold biomaterial should have biological cues such as cell adhesive ligands to enhance cell attachment or physical cues such as topography to influence cell morphology and alignment (Chan and Leong 2008). However, many scaffolds are fabricated from synthetic polymers due to their mechanical strength, so are often coated with natural based polymers/ECM proteins. For example Li et al. coated electrospun polycaprolactone fibres with gelatine which improved biological activity compared to the uncoated fibres (Li et al. 2008). Gelatine is effective at enhancing cell adhesion because it contains abundant Arg-Gly-Asp (RGD) sequences which are the cell attachment sites recognised by many integrins. The presence of RGD sequences therefore facilitates cell adhesion and spreading (Xing et al. 2014). In addition, Attia et al. (2011) coated synthetic polyurethane fibres with a variety of ECM proteins including fibronectin, collagen type I and vitronectin and found that fibronectin demonstrated the greatest cell attachment, and influenced cell spreading and alignment.

Fibronectin is a multifunctional glycoprotein present in plasma in a soluble form and in the ECM. It is expressed by many cell types and contributes to cell adhesion, migration, proliferation and tissue development (Attia et al. 2011). Some scaffolds can incorporate biomolecules such as growth factors, where the scaffold serves as a delivery vehicle to the cells to accelerate and enhance tissue regeneration (Chan and Leong 2008). Growth factors are secreted by cells and act as guidance signals for cell behaviour including cell proliferation, migration, differentiation and tissue regeneration (Zhang et al. 2016). Through utility of encapsulation methods within scaffold fibres, biomolecules with retained bioactivity can be released in a controlled manner (Chan and Leong 2008). However, growth factors often have a short half-life and the ability to deliver the growth factor specifically to the cells can be a drawback during tissue regeneration (Zhang et al. 2016). Some examples of growth factor encapsulation within fibres includes a study by Wang and Wang (2017). They fabricated electrospun nanofibrous scaffolds and incorporated growth factors including recombinant human vein endothelial growth factor which subsequently enhanced cell viability of human umbilical vein endothelial cells. Zhang et al. also prepared coaxial electrospun fibres with the encapsulation of basic fibroblast growth factor (bFGF) within the core of the fibres (Zhang et al. 2016). The fibres were able to achieve controlled release of growth factors with different rate and amounts. Table 3 describes the functions of the native ECM tissues and the features possessed by scaffolds to recreate the biological and biomechanical cues of the ECM.

**Table 3:** Summary of functions of the ECM in native tissues and how scaffolds in engineered tissues mimic the ECM

ECM in native tissues	Scaffolds in engineered tissues
Provides structural support for cells to reside	Porous, interconnected structure to support cell attachment, growth, migration and differentiation
Contributes to the mechanical properties of the tissues	Provides the shape and mechanical stability to the tissue defect and gives the rigidity and stiffness to the engineered tissues
Provides bioactive cues for cells to respond to their microenvironment	Can have binding sites such as RGD sequence and surface topography which interacts with cells actively to facilitate activities such as proliferation and differentiation
Acts as the reservoirs of growth factors and potentiates their actions	Serves as a delivery vehicle and reservoir for applied growth factors
Provides a flexible physical environment to allow remodelling in response to dynamic tissue processes	Provides a void volume for vascularisation and new tissue formation during remodelling. Porous microstructure allows nutrients and metabolites to diffuse. Degradation mechanisms and rates can be controlled

## Scaffold materials (synthetic vs natural polymers)

Scaffold materials can be composed of synthetic or natural polymers which offer different properties such as high porosity, tailored pore sizes, biodegradation, mechanical strength dependent on their composition, structure and arrangement of their constituent macromolecules (Dhandayuthapani et al. 2011). They are easy to process and can easily incorporate bioactive molecules to subsequently mimic the ECM structure. Using synthetic and natural polymers as constructs for tissue engineering has its advantages and disadvantages as reviewed by Bhatia (Bhatia 2016). However, synthetic polymers are more stable than natural polymers and therefore have a longer shelf life, can be readily sterilised, and are less temperature sensitive than natural polymers. They are also more cost effective than natural polymers, can be produced under controlled conditions, and exhibit predictable and reproducible mechanical and physical properties, such as tensile strength, elastic modules and degradation rate. Examples of synthetic polymers used for scaffolds include: poly (vinyl chloride), poly(caprolactone), poly (lactic acid), poly (lactic-co-glycolic acid) and poly (ethylene terephthalate). Natural polymers are derived from living sources such as the human body or animals. As natural derivatives, they have bioactive properties, which allow them to have better interactions with cells compared to synthetic polymers (Dhandayuthapani et al. 2011) as well as to enhanced biocompatibility and less toxicity. Examples of natural polymers used for scaffolds include gelatine, collagen, fibrinogen and elastin.

## Scaffold fabrication techniques

There are a variety of approaches to fabricate scaffolds for tissue engineering which should consider variables such as biocompatibility, biodegradability, mechanical strength, pore size, scaffold architecture and manufacturing technology (Plunkett and O’Brien 2011). Each approach has its own advantages and disadvantages in preferred tissue engineering applications, whilst different types of cells prefer different scaffold structures. The array of scaffold fabrication techniques include: (1) solvent-casting and particulate-leaching (2) melt moulding, (3) freeze drying, (4) thermally induced phase separation (5) gas foaming, (6) electrospinning and (7) 3D printing.

### Solvent-casting and particulate-leaching

**Fig. 2:**
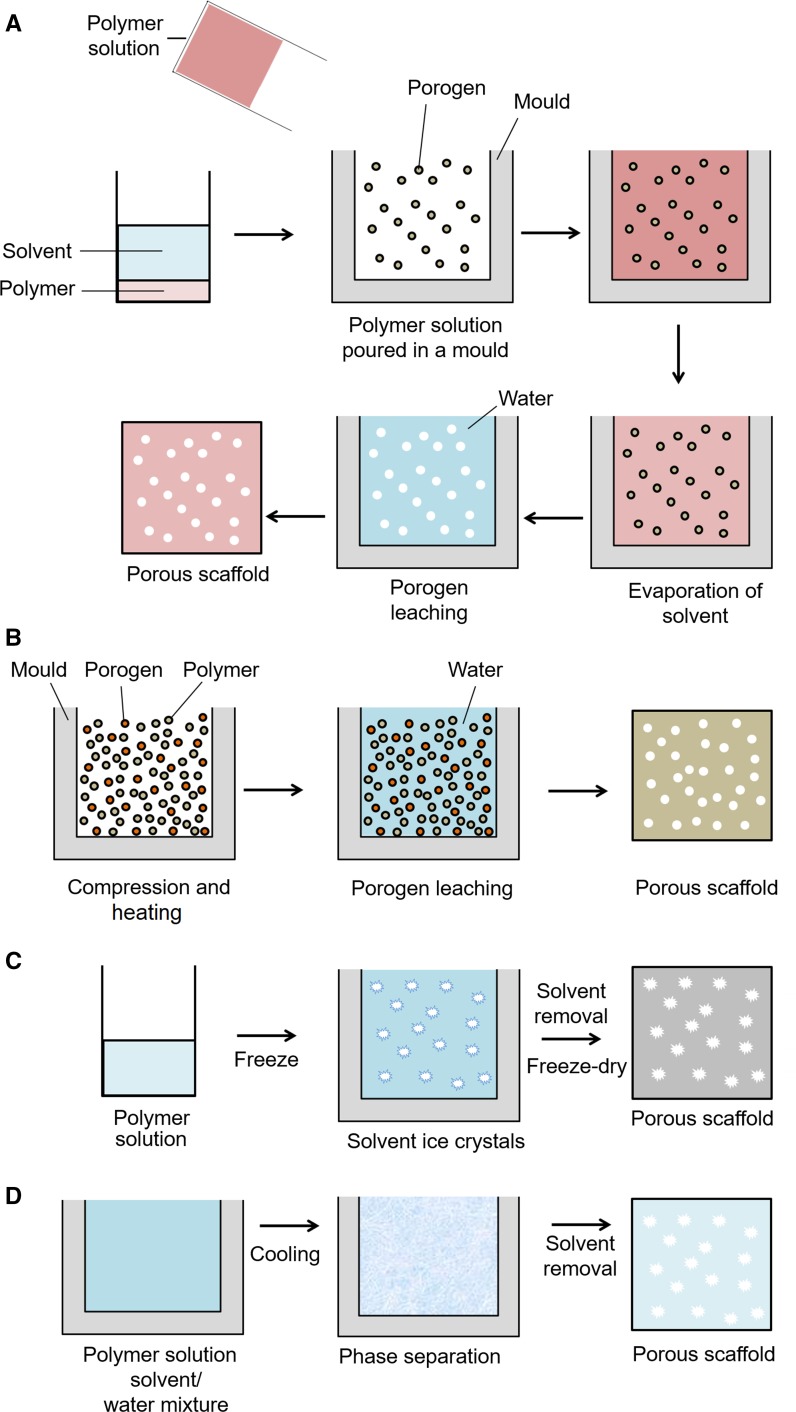
**a** Solvent casting/particulate leaching process: The polymer is dissolved in a solvent and poured into a mould containing a porogen. Upon solvent evaporation the polymer matrix with the porogen remains. The porogen is eliminated by immersing in aqueous media, thus producing a porous structure. **b** Melt Moulding: Moulds are filled with a powdered polymer and a porogen, pressure and heat is applied. The porogen is leached out by washing with water to leave behind a porous scaffold **c** Freeze-drying: The polymer solution is cooled to a frozen state using liqud nitrogen. The solvent forms ice crystals causing the polymer molecules to aggregate in between. The solvent is removed by sublimation of the solvent and reduced pressure, this leaves behind a porous scaffold. **d** Thermal induction phase separation: Polymer powder is dissolved in a solvent mixture and heated. The polymer solution is s cooled, and phase separation takes place due to the thermodynamic instability. The solvent is removed by freeze drying leaving behind a porous scaffold made up of polymer-rich/poor phases

Solvent-casting and particulate-leaching involves a simple and cost-effective process of mixing a polymer solution with salt particles of a specific diameter to produce a porous scaffold. As shown in Fig. 2a, the polymer is firstly dissolved in an organic solvent and then poured into a mould containing a porogen such as sodium chloride (Sampath et al. 2016). The solvent then evaporates leaving behind a polymer matrix with salt particles within. The construct is then immersed in water where the salt particles leach to fabricate a porous structure (Wosek 2015). An advantage to this technique is that the pore size and overall porosity can be tuned by changing the particle size (Annabi et al. 2010), which is fairly reproducible. Solvent casted/particulate leached scaffolds can be used for applications such as bone tissue engineering and have been shown to support osteoblastic cell growth and mineral deposition (Thadavirul et al. 2014). Constructs have been fabricated from polymers such as Food and Drug Administration (FDA) approved polycaprolactone which exhibits excellent biocompatibility and mechanical strength (Thadavirul et al. 2014). Highly porous scaffolds can be produced, which is important for mass transport requirements for cell nutrition, porous channels for cell migration and surface features for cell attachment (Hollister 2005). However, an increased porosity can compromise the structural stability of the biomaterial, and therefore a balance is needed between the mechanical and mass transport function of the construct to create an optimal scaffold system (Loh and Choong 2013). A drawback of this method is that new tissue formation of often limited to the surface of the construct with minimum cell growth near the centre of necrotic zones in the centre of the construct (Sopyan et al. 2008). Furthermore solvent residues from the porogen or solvent could be harmful/toxic to cells (Sopyan et al. 2008).

### Melt moulding

Melt moulding can be used to create polymeric scaffolds. The process involves filling moulds with a powdered polymer, and porogen compounds above the polymer’s glass transition temperature at an elevated pressure (Janik and Marzec 2015). These combine to form a scaffold in the shape of the mould. The porogen is leached out by washing with water to yield a porous polymer scaffold as shown in Fig. 2b. The constructs exhibited high porosity and bone formation within the scaffold for bone tissue engineering applications. The advantages of this method are that it is convenient, economical and does not require toxic solvents. Furthermore, polymer scaffolds can be rapidly produced of various shapes, sizes and tailored pore size dependent on the porogen used (Janik and Marzec 2015). The limitations of this method involve difficulty in leaching out residual porogens, which could affect tissue culture (Janik and Marzec 2015). In addition, if incorporating bioactive compounds into the construct, the high temperatures used may destroy the molecules.

### Freeze-drying

Freeze drying is a method used to make porous materials for regenerative medicine applications (Offeddu et al. 2015). Figure 2c shows the freeze-drying process of a scaffold. The first stage of freeze-drying involves cooling a polymer solution to a frozen state. The solvent then forms ice crystals forcing the polymer molecules to aggregate in between (Zhu and Che 2013). The solvent is removed by reducing the pressure and subliming the solvent. This leaves behind a dry polymer scaffold with a highly porous interconnected porous microstructure. Jin et al. (2015) fabricated polycaprolactone/chitosan composite scaffolds via freeze drying for bone regeneration applications. An advantage to the freeze-drying method is that water can be used as the solvent instead of an organic solvent which is more suitable for biomedical applications (Lu et al. 2013). A disadvantage to this method is that, although a highly porous construct can be fabricated, it is more difficult to control the pore size.

### Thermally induced phase separation

Phase separation is a thermodynamic process involving the separation of phases due to physical incompatibility to create scaffolds for tissue engineering (Chen et al. 2013). As shown in Fig. 2d, the first step in scaffold preparation is to make a uniform and homogeneous polymer solution. The polymer is dissolved in a solvent and becomes thermodynamically unstable by heating the mixture for a certain period and temperature, in addition to subsequent cooling. The thermal energy helps induce the phase separation separating the solution into a polymer rich and polymer lean phase (Akbarzadeh and Yousefi 2014). The solvent is then removed by either freeze-drying or freeze-extraction (Akbarzadeh and Yousefi 2014). The polymer-rich phase will solidify to form a 3D matrix while the polymer-poor phase will become the void space (Chen et al. 2013). Yen et al. (2009) fabricated nano-porous polycaprolactone scaffolds which demonstrated controlled drug release for drug delivery applications. In addition, Conoscenti et al. fabricated highly porous, well defined pore sized poly(l-lactic acid) scaffolds for bone engineering applications, and demonstrated the scaffolds were able to support chondrocyte differentiation (Conoscenti et al. 2017). An advantage to this technique is that by easily changing parameters such as polymer type, solvent/non-solvent ratio, polymer concentration, heating temperature and time, and cooling rate; porous constructs can be fabricated with specific morphologies for a particular application (Akbarzadeh and Yousefi 2014). Thermally induced phase separation is a useful technique for developing scaffolds with well-defined pore shape and pore size and can be combined with other fabrication methods to control the final 3D structure (Chen et al. 2013). However, the drawbacks of this technique includes minimal control over fibre orientation and diameter, long fabrication time, and lack of mechanical properties.

### Gas foaming

Gas foaming eliminates the use of harsh chemical solvents by creating highly porous polymer scaffolds by using high pressure carbon dioxide. Solid discs of a polymer such as polyglycolide and poly-l-lactide are first formed by compression moulding at high temperatures (Loh and Choong 2013). High pressure carbon dioxide (800 psi) is then applied to saturate the polymer within an isolated chamber over a certain period. Rapid depressurisation causes thermodynamic instability and leads to form nucleated gas cells creating pores inside the polymer matrix (Sampath et al. 2016). Scaffolds sourced from poly(d,l-lactic-co-glycolic acid)/nano-hydroxyapatite (PLGA/HA) have been fabricated by this technique for bone tissue engineering and have shown to exhibit efficient osteoblast growth and activity for future bone regeneration applications (Kim et al. 2006). A disadvantage to this technique is the inability to ensure pore connectivity and control pore sizes by gas forming. In addition high temperatures during disc formation can inhibit the use of bioactive molecules in the scaffolds (Loh and Choong 2013).

### Electrospinning

**Fig. 3:**
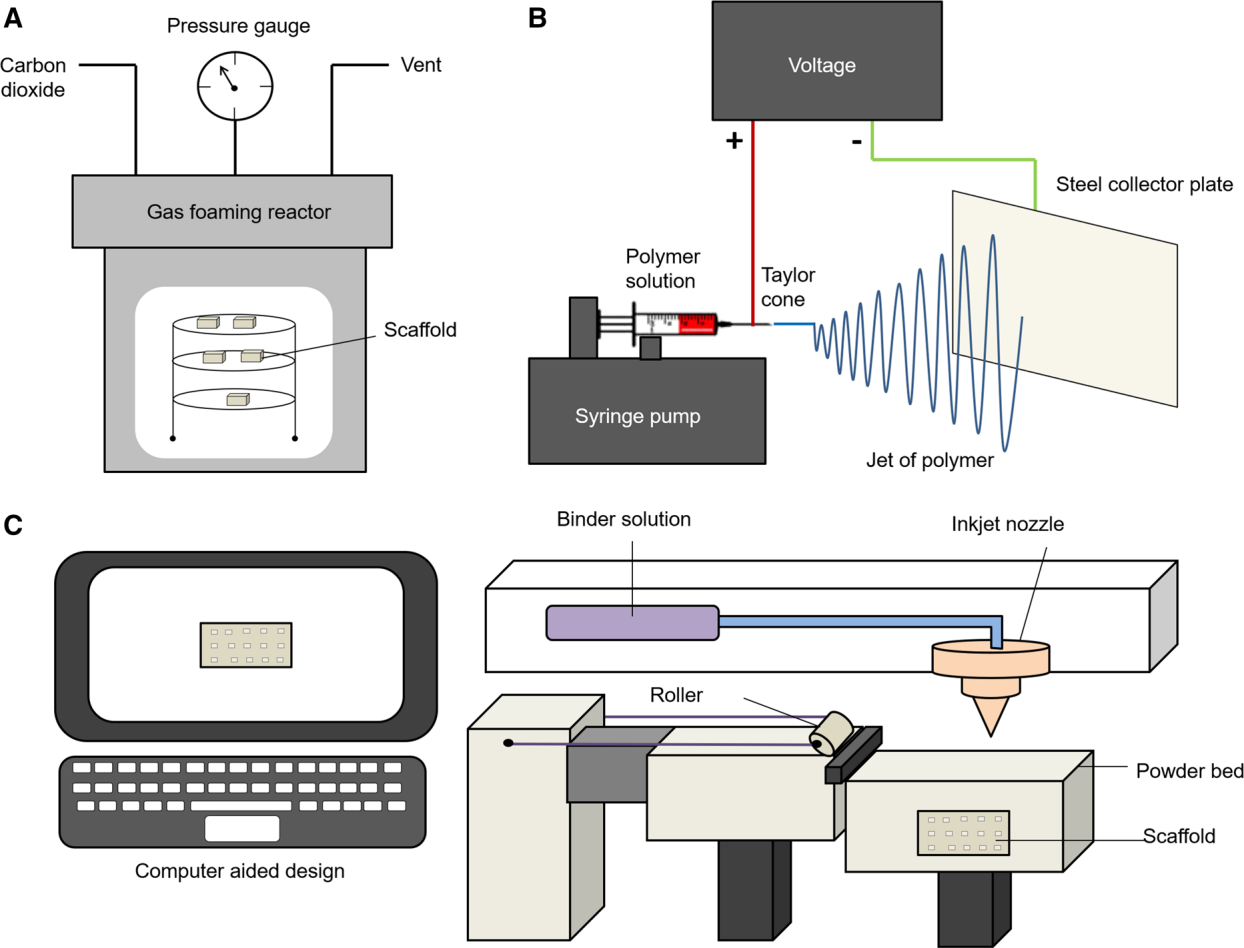
**a** Gas foaming: The polymer is firstly compressed and moulded at a high temperature. The polymer is placed inside a gas foaming reactor and exposed to high pressure carbon dioxide to saturate the polymer. Rapid depressurisation leads to the formation of nucleated gas cells creating pores in the polymer scaffold. **c** 3D printing: Uses computer aided design to create a digital template to print. A thin layer of powder is placed on the powder bed and spread using a rolling mechanism. The printing machine reads the design of the template and the inkjet nozzle selectively lays down the binder solution into a powder bed. The layering is repeated to create a 3D model. The excess unbound powder is removed leaving behind the construct. **b** Electrospinning: A polymer is dissolved in a solvent and the polymer solution is placed in a syringe onto a syringe pump. A voltage is applied to the polymer solution the tip of the polymer drop at the end of the needle is stretched into a Taylor cone. This then becomes unstable and produces a polymer jet which is attracted to the oppositely charged collecting plate

Electrospinning is a simple technique compared to the others due to the ability to easily control specific parameters to fabricate a scaffold on the nano/micro scale to mimic the fibrous structure of the native extracellular matrix. Harrington et al. (2014) demonstrated how an electrospun polymeric scaffolds can be used to model decellularized lung extracellular matrix. Electrospun scaffolds also offer a high surface area, tuneable porosity, flexibility to cater to a different sizes and shapes, and the ability to control the fibre composition to achieve the specific properties or functionality (Bhardwaj and Kundu 2010). The basic electrospinning set up consists of a syringe pump, polymer solution, needle, voltage supply and collecting plate (Haider et al. 2015), as shown in Fig. 3b.

The electrospinning process is usually conducted at room temperature and begins when a high voltage is applied to the polymer solution and the polymer droplet at the needle tip is held by surface tension. At a critical voltage the surface tension of the liquid is overcome causing the droplet to elongate into a Taylor Cone. A continuous fine fibre jet is ejected from the tip of the Taylor Cone and is accelerated towards the oppositely charged grounded collecting plate. As the fibre travels through the air the solvent evaporates and solid polymer fibres are deposited on grounded collector as a scaffold (Haider et al. 2015; Pillay et al. 2013). Although electrospinning appears to be a simple process, a disadvantage to this technique is that several parameters can affect the fibre morphology and need to be optimised to produce smooth uniform fibres such as: voltage, flow rate, polymer concentration, solvent, relative humidity, distance from the needle tip to the collecting plate, and temperature. Without the optimal conditions, fibres produced may be too thick or thin, or can become beaded leading to a non-uniform structure or may not spin at all.

Electrospinning is a versatile technique and has been used for many tissue engineering applications, including (1) skin tissue, using poly(lactic-co-glycolic acid) (PLGA) scaffolds (Ru et al. 2015), (2) bone tissue using polycaprolactone (PCL), PLLA, silk, and collagen (Khajavi et al. 2016), (3) corneal tissue, using poly-l-lactic acid (PLLA) PCL, PLGA, gelatine, silk, and collagen (Kong and Mi 2016), (4) cardiac tissue using PLGA scaffolds (P Prabhakaran et al. 2011), (5) drug delivery (Mirjalili and Zohoori 2016) (6) wound healing (Askari et al. 2016)and (7) detection of metal ions (Kim et al. 2017).

### 3D printing

3D printing uses an inkjet printing liquid binder to make a 3D object from digital model data shown in Fig. 3c. The first step of 3D printing involves modelling a virtual model using computer-aided design where the machine uses this as a template to print (Sampath et al. 2016). A thin layer of powder is deposited onto a powder bed and is spread and levelled onto a building platform using a roller system (Do et al. 2015). The machine reads the design of the digital model data and a printer nozzle selectively lays down liquid binder solution into a powder bed to form a 2D pattern (Sampath et al. 2016). This process is repeated layer by layer to produce a 3D model. Once the binder solution and powder are combined the excess unbound powder is removed (Do et al. 2015).

Sun et al. (2016) fabricated highly porous collagen/silk constructs using 3D printing for applications in bone tissue engineering. They found that bone mesenchymal stem cells were able to maintain their viability, proliferate and deposit ECM proteins efficiently. In addition, the 3D printing technology was found to be simple, easy to operate, was fast at printing and can print and assemble bioactive tissue. However, attention needs to be paid when selecting the composition ratio of the material for printing as unsuitable proportions or incompatible materials can result in interference with the spray nozzle or block the print head resulting in unstable three-dimensional scaffolds and poor performance (Sun et al. 2016). The main advantages of 3D printing include the ability to fabricate versatile scaffolds with complex shapes and the ability to imitate the extracellular matrix (Do et al. 2015). However, this can be limited by the use of printable materials that have the stability and desired properties for 3D printing, often alternative material methods processing methods are required to work with materials not easily printed (Chia and Wu 2015). Furthermore, incorporating bioactive molecules can be a challenge as they may be sensitive to the printing environment (Wu and Hsu 2015); particularly if the printing processes involve a solvent or extreme temperature the proteins folding may be affected, or they can be denatured (Wu and Hsu 2015). Production time for scaffold fabrication can become lengthy as the scaffold design becomes more precise and intricate (Do et al. 2015). Other methods of 3D printing reviewed by Mota et al. (de Azevedo Gonçalves Mota et al. 2016) include selective laser sintering, stereolithography, fused filament fabrication,solvent casting 3D printing and more recently, digital light processing (Düregger et al. 2018).

## Bioreactors

Bioreactors complement the use of scaffolds in tissue engineering, and can be described as devices that utilise mechanical methods to influence biological processes (Plunkett and O’Brien 2011). Cell-seeded porous scaffolds have been placed in a range of different bioreactors including (1) orbital shakers, (2) spinner flasks, (3) rotating wall vessels, (4) perfusion bioreactors and (5) microfluidic devices, to aid the production of functional 3D tissues. The key features for an ideal bioreactor system are given in Table 4.

**Table 4:** Key features required for an ideal bioreactor system

Features	Description
Leak proof	Reduces risk of contamination, and loss of reagents
Optically transparent	Allows in situ real time monitoring
Easy to assemble	Less training required, rapid experimental set-up
Ability to monitor microenvironment	Provide data on culture conditions such as pH, oxygen, carbon dioxide, metabolites
Allows use of different flow types/rates	Different flow rates/types are required for different cell types/applications
Allows easy insertion and retrieval of scaffolds	Allows 3D cell culture and post analysis.
High throughput	Quicker data acquisition
Flexible configuration	Modular interconnected systems allow co-culture and cell–cell signalling
No air bubble formation	Presence of air bubbles can disrupt the flow rate and disturb cells

They maintain a desired uniform cell concentration within the scaffold during cell seeding (Salehi-nik et al. 2015) which facilitates adequate cell–cell interactions (Kumar 2016). Exposure to medium fluid flow can be used to mimic physiologic delivery of oxygen, nutrient supply, chemical signals and continuous waste removal from 3-D tissue engineered constructs and has been shown to provide significantly higher mass transfer rates compared to static cultures (Rangarajan et al. 2014). The fluid shear stress caused by mixing or perfusion of culture medium will expose cells to mechanical stimulation (Gaspar et al. 2012) that can mimic stimulants such as interstitial flow which can affect cellular alignment and differentiation (Ng and Swartz 2006). Bioreactors have also been shown to enhance the rate of proliferation and reduce necrotic core formation in scaffolds. compared to static cultures (Khang 2017). Bioreactors are limited by the lack of specific guidelines available in terms of which flow rate/speed to use or volume of culture medium, as different cells have different cell culture requirements (Ismadi et al. 2014). Many bioreactor systems also do not incorporate the ability to non-invasively monitor the microenvironment in real time, which means important parameters such as oxygen, pH, temperature cannot be controlled. Bioreactor systems should be chosen based on their specific application, some of which permit turbulent or laminar flow, others are more suited for suspension cultures or adherent cell types, and some bioreactors are necessary for larger scale culture.

### Spinner flask

**Fig. 4:**
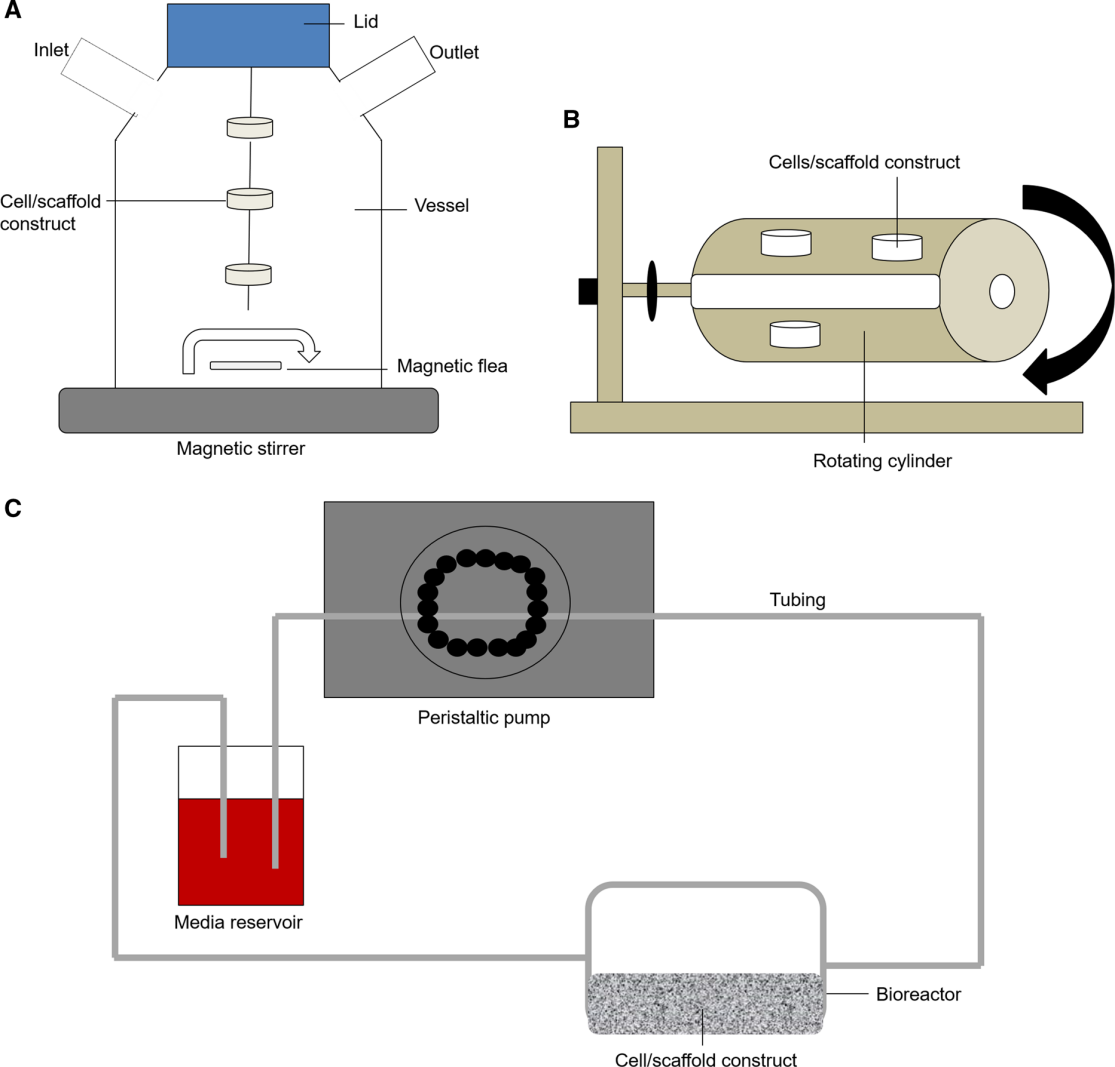
**a** Spinner flask: Scaffolds are threaded through needles within a glass container. A magnetic stirrer is used to stir the medium throughout the construct. **b** Rotating wall vessel: Scaffold constructs are placed in a cylindrical bioreactor filled with medium. The bioreactor is rotated along the horizontal axis to stir the medium **c** Perfusion bioreactors: Medium is pumped around a circuit by a peristaltic pump. The media passes through the bioreactor containing the scaffold construct. The set up can be recirculating or single pass

The spinner-flask bioreactor was developed to create a convective flow and produce hydrodynamic forces that help mass transport throughout cell seeded scaffolds (Gaspar et al. 2012). Spinner flasks consist of a cylindrical glass container in which growing tissues are suspended and a stirring element such as a magnetic stirrer is placed at the bottom of the tank ensuring the mixing of the culture medium (Sucosky et al. 2004). The scaffolds are in fixed positions, threaded in needles attached to the cap of the container (Gaspar et al. 2012), shown in Fig. 4a. The mixing mechanism of this bioreactor has been shown to improve cellular distribution and differentiation in scaffolds (Stiehler et al. 2009). Spinner flasks are commonly used for bone tissue engineering as they can mimic some aspects of the native bone environment. However, spinner flasks are thought to only permit the extracellular matrix production at the scaffolds surface and mixing the media can create turbulent shear at the surfaces which can be unfavourable to cell growth and tissue formation (Gaspar et al. 2012).

Spinner flasks can be set up in batch and continuous cultures. Batch mode is a closed type of cultivation system that does not allow for the addition of fresh medium or the removal of waste, this can limit the product yield, whilst overcoming the risks of contamination. This method is also limited both in scale and length of culture due to a build-up of metabolites and waste that occurs over time. Continuous cultures allow the removal of waste, but this exposes the culture to a maximum chance of contamination. Fed-batch is an intermediate and is a semi closed type, it allows the addition of fresh nutrition but no removal of waste which produces a medium yield.

### Rotating wall vessel

Rotating wall vessels consist of cylindrical bioreactors which are filled with culture medium and rotated along a horizontal axis as shown in Fig. 4b. The physiological low fluid shear stress environment is usually used for suspension cultures, where the cells can aggregate based on their natural cellular natural cellular affinities, form 3-D structures and acquire properties of highly differentiated cells (Skardal et al. 2010). Studies have been performed to investigate the effects on dynamic flow in a 3D environment on bone cell biology and bone formation in vitro. Adherent cell lines can be cultured on scaffolds, however these can experience repeated collisions with the bioreactor wall which has been shown to limit achievable cell density (Yu et al. 2004).

### Perfusion bioreactors

Perfusion bioreactors are used to provide a flow of medium through or over a cell population, in order to help push the oxygen and nutrients through the pores of 3D scaffolds (Salehi-nik et al. 2015). Different types of perfusion bioreactors are available, some of which are commercially available whilst others are produced in-house for various types of applications. Figure 4c shows a standard set up of a perfusion bioreactor system. Perfusion bioreactors are very versatile and generally can be set up in different configurations, including a closed set up where the media recirculates to provide media containing naturally produced growth factors, or single pass set up where only fresh media is supplied to the cells avoiding the accumulation of metabolites. Flow rates should be optimised when setting up perfusion bioreactor systems, as cells can be damaged at high flow rates, or may not have sufficient nutrient and oxygen supply at low flow rates. Flow can be used to deliver shear stress such as unidirectional laminar, pulsatile laminar, turbulent and oscillating flow. Perfusion bioreactors have been used to provide shear stress to induce human mesenchymal stem cells (Lembong et al. 2018; Bhaskar et al. 2017), cardiovascular engineering (van Haaften et al. 2018),

### Quasi-Vivo^®^

The Quasi Vivo^®^ (QV) is a perfusion bioreactor system commercially available in different formats and configurations. “Quasi” is derived from the Latin definition of ‘resembling, but not actual’, and “vivo” from the same derivation meaning ‘living thing’, together the name represents a system that can create conditions that are very similar to physiologically relevant conditions in living organisms.

**Fig. 5:**
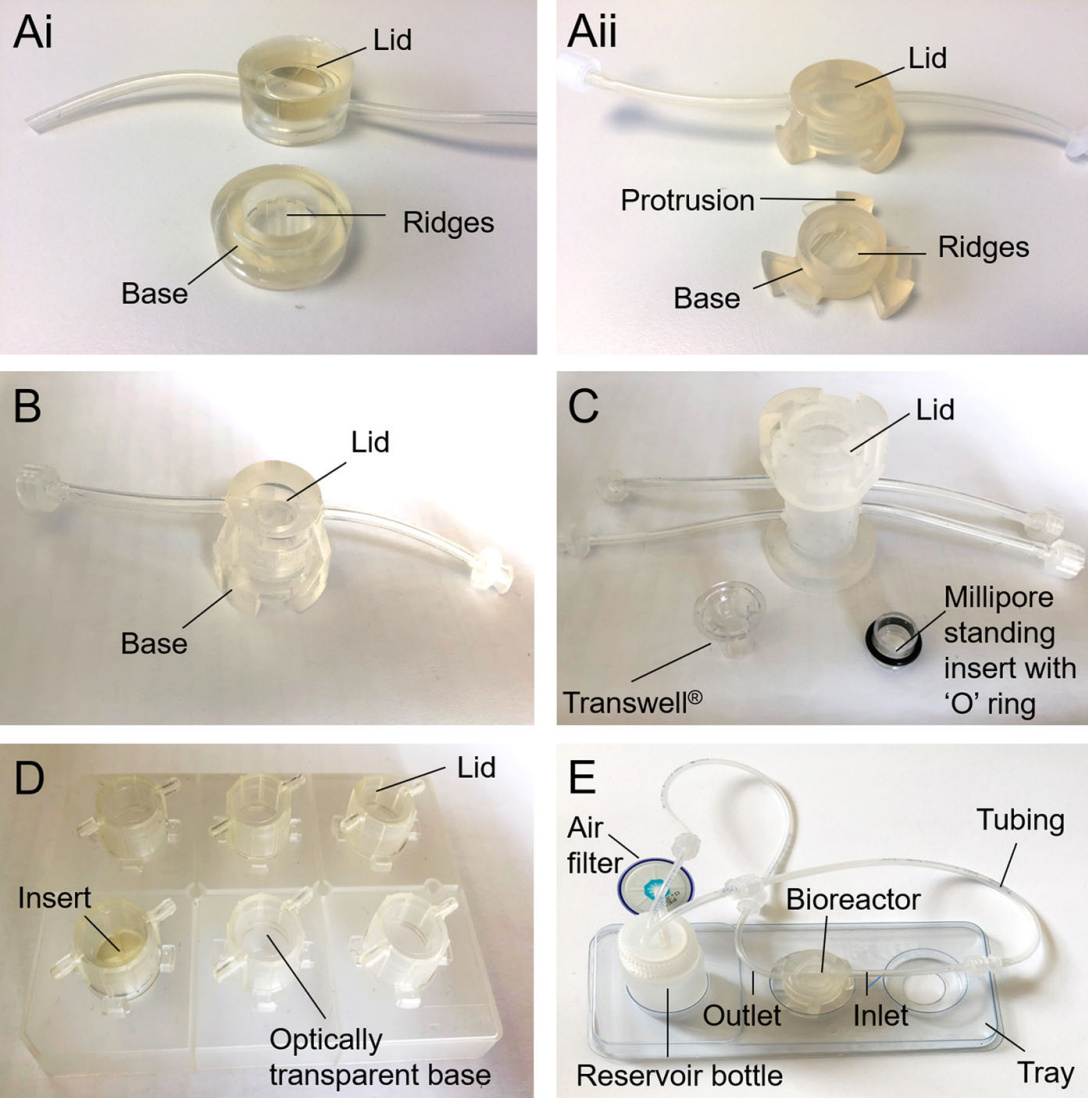
**a** Photographs of showing the slight difference in structure of the original McmB and the patented Quasi Vivo^®^ chamber (i) McmB (ii) Quasi Vivo^®^500. **b** Quasi Vivo^®^500 chamber for submerged cultures. **c** Quasi Vivo^®^600 chamber, compatible with commercially available Transwells^®^ for air liquid interface applicattions, and also Millipore standing inserts when secured by an ‘O’ ring for liquid–liquid barrier applications. **d** Quasi Vivo^®^900 for submerged cultures. The chambers have an optically transparent window at the base of the chamber to allow live cell imaging, and the trays are made of acrylic to help reduce non-specific binding of compounds. **e** Quasi Vivo^®^ system with reservoir, tubing and bioreactor

There are a variety of different QV bioreactor systems to suit different tissues and applications including QV500, QV600 and QV900 shown in Fig. 5. Some of the advantages of the QV systems is that all of the systems can be set up to either provide a single flow of fresh medium, or recirculating medium which removes the risk of shock or disturbance to the cells during feeding (Przyborski 2017). In addition, recirculating media enables the production of conditioned media containing a cocktail of growth factors and cytokines. All of the chambers can accommodate an adjustable laminar flow rate and chamber pressure to suit the specific requirements of different tissue types (Przyborski 2017). The Quasi Vivo^®^ systems are known for their simplicity, ease of use, and variety of published studies showing the enhanced cell activity using these bioreactors. The ability to easily insert/retrieve scaffolds from the bioreactor is useful for post analysis i.e. immunohistochemistry. The QV systems all have a flexible modular configuration, the individual bioreactors can be interconnected to allow multiple or the same cells types to be cultured in separate chambers. This can enable cross talk between the tissues which is important when recreating specific organ interactions.

QV500 applications include cardiovascular stem cell differentiation (Pagliari et al. 2014), fluid shear stress on hepatocytes (Rashidi et al. 2016), a interconnected blood brain barrier model (Miranda-Azpiazu et al. 2018) and nanotoxicity with endothelial cells (Ucciferri et al. 2014). The QV600 has been used for the preparation of lung models, where it has been has shown accelerated proliferation of epithelial cells (Chandorkar et al. 2017), as well as gastrointestinal tract and blood brain barrier models. The most recently developed QV900 consists of multiple optically transparent bioreactors integrated into a multi-well tray that enhance monitoring of parameters though microscopy, Fig. 5d. The QV900 is fabricated from acrylic to reduce non-specific binding of molecules and compounds for drug development applications. (Stosik et al. 2018) recently used the system for drug exposure, comparing the CYP activity in primary human hepaotcytes in flow conditions vs static. Nithiananthan et al. used the QV900 to investigate the effect interstitial fluid flow on fibroblast response (Nithiananthan et al. 2016).

### Microfluidics

Perfusion bioreactors can also come in the form of microfluidic devices to miniaturise macroscopic systems for higher throughput of biological experiments. In addition, they enable studies of cell behaviour of organisms with precise and localised application of experimental conditions which are difficult to achieve using macroscopic tools (Velve-Casquillas et al. 2010). Microfluidic devices include organ-on-chips where specific cell types are cultured and continuously perfused within micrometre-sized chambers to model physiological functions of a particular tissue or organ (Bhatia and Ingber 2014).

Devices are often fabricated from PDMS using rapid, simple, and inexpensive techniques such as soft lithography, which involves the replication of a topographically defined structure on a master in a soft elastomer (Tang and Whitesides 2010). The designs of microfluidic devices are very flexible and can therefore cater to for variety applications, however each device is highly specific to single experimental configurations. Due to the very small nature of the platform, only a low number of cells and reagents are required which is more cost efficient. Live cell imaging and real time on chip analysis can be performed with direct coupling to down-stream analysis systems (Halldorsson et al. 2015).

Some of the drawbacks of the microfluidic devices include the non-standard culture protocols entail complex operational control and chip design. In addition, the reduction in scaling can provide challenges in adapting biological protocols to fit experiments based in a microsystem, such as the media and cell concentration (Velve-Casquillas et al. 2010). Small working volumes for seeding and reagents can also be challenging for subsequent analytical chemistry, complex operational control and chip design (Halldorsson et al. 2015). Furthermore, many in vitro models now push the use of scaffolds to create the native microenvironment, however loading and retrieving scaffolds from the microfluidic devices can be difficult, and even when inserted the scaffold area would be very small. Dongeun Huh describes the fabrication of a PDMS microfluidic device to replicate the microarchitecture and dynamic microenvironment of the alveolar–capillary unit of the living human lung (Huh 2015). A variety of commercially available perfusion bioreactors are available for different applications and come with their own advantages and limitations.

## Future outlook

## Monitoring of culture conditions and tissue constructs

Many complex in vitro models have been developed for specific tissue engineering and regenerative medicine applications. However, one of the challenges is continuous monitoring of cellular activities within 3D, generally opaque thick structures (Ozcelik et al. 2014). Many of the developed in vitro models are limited by the ability to monitor cell culture conditions in a non-invasive manner. With the lack of ability to monitor the tissue regeneration processes in situ, it can limit our understanding of optimal conditions required for growth (Harrington et al. 2013). Therefore, novel techniques for monitoring in vitro cultures at all stages of tissue growth, repair and regeneration in a more insightful, non-invasive and quantitative manner is imperative (Papantoniou et al. 2014; Kotecha et al. 2013).

With non-invasive in situ monitoring in real time we can monitor cell growth, cellular differentiation and tissue morphogenesis (Kotecha et al. 2013), and develop more reliable tissue engineered constructs that are more physiologically relevant models for disease and drug testing. Moreover, non-invasive monitoring can provide real time functional read outs, without having to disturb the cellular microenvironment or introduce potential contamination. Currently widely used methods of monitoring tissue engineered constructs include destructive end point determination and biochemical or histological methods to determine cell number, viability and tissue growth throughout the construct (Papantoniou et al. 2014). Therefore simple and readily applicable non-destructive methods of monitoring changes in cell metabolism, viability and tissue deposition particularly within long term cultures would be invaluable and could point out adverse responses during the early stages of culture (Boubriak et al. 2006).

## Biosensors

Biosensors can be used for direct real-time monitoring of processes within engineered tissues (Ozcelik et al. 2014). Biosensors can be defined as “a self-contained analytical device that combines a biological component with a physicochemical component for the detection of an analyte of biological importance” (Hasan et al. 2014). By detecting cellular analytes, electrical activity, physical and chemical signals transmitted by cells, biosensors can provide insights into cellular activities and responses in real time (Perestrelo et al. 2015). When designing robust biosensors they should meet several requirements such as being able to detect trace amounts of biomarkers within complex biological environments such as cell culture medium, which usually contains a plethora of nonspecific proteins and interfering compounds (Shin et al. 2017). In addition, the robust biosensor systems should be able to have continual monitoring capability every few hours or days for kinetics analysis of biomarkers over extended periods (Shin et al. 2017). Biosensors are made up of three main components, 1) a detector to detect the stimulus, 2) a transducer to convert the stimulus to output signal, 3) a signal processing system to process the output and present it in an appropriate form (Hasan et al. 2014). Hasan et al. (2014) reviews the different kind of biosensors and its components. The sensing component or bioreceptor includes enzymes, microbes, cells, nucleic acids, and antibodies. The different types of transducers are listed in Tables 5 and 6. The applications of biosensors include sensing small molecules such as glucose, hydrogen peroxide, adenosines, functional protein molecules, pathogenic microbes.

**Table 5:** Different types of transducers and the measured property for tissue engineering applications

Type of transducers	Measured property	Compatible with bioreactor?	Example/reference
Electrochemical	Potentiometric, Amperometric, Conductometric, Nanotechnology, Bioelectronics	Yes	Electrochemical immunosensors integrated into bioreactors for continual monitoring of cell secreted biomarkers Riahi et al. (2016)
Protein	Immunosensor	Yes	Glucose monitoring in living cells using single fluorescent protein-based sensors Hu et al. (2018)
Electrical	Surface conductivity, Electrolyte conductivity	Yes	Electrical Impedance Spectroscopy cell monitoring in a miniaturised bioreactor Martínez-Teruel et al. (2013)
Optical	Fluorescence, Adsorption & Reflection	Yes	Low-cost calibration-free pH sensing with disposable optical sensors (Ge et al. 2012)
Light	Bioluminescence	Yes	Real-Time Bioluminescence Imaging of Cell Distribution, Growth, and Differentiation in a Three-Dimensional Scaffold Under Interstitial Perfusion for Tissue Engineering Vila et al. (2016)

**Table 6:** Different types of biosensors

Measurement type	Transducer	Transducer analyte
Potentiometric	Ion-selective electrode	K^+^, Cl^−^, Ca^2+^, F^−^,
	Glass electrode	H^+^, Na^+^,
	Gas electrode	CO_2_, NH_3_,
	Metal electrode	Redox species
Amperometric	Metal or carbon electrode	O_2_, sugars, alcohols,
	Chemically modified electrodes	Sugars, alcohols, phenols, oligonucleotides
Conductometric	Interdigitated electrodes	Urea, charged species, oligonucleotides
	Metal electrode	

### Electrochemical biosensors

Electrochemical sensors operate by reacting with an analyte of interest to produce an electrical signal proportional to the analyte concentration (Hammond et al. 2016). Different types of electrochemical biosensors measurements include potentiometric, amperometric and conductometric which can detect a variety of analytes, see Tables 5 and 6. One of the key advantages of electrochemical biosensors is their simplicity. Inexpensive electrodes can be easily integrated with simple electronics to perform rapid measurements in miniaturised easy-to-use portable systems. Miniaturisation is important because biological samples are often available in small amounts, and tissue damage must be minimised in cases of in vivo monitoring (Săndulescu et al. 2015). Being able to determine the concentration of an analyte within a complex sample at the point-of-care and in near real time with short response times is extremely attractive for medical diagnosis, monitoring of existing conditions and environmental monitoring (Hammond et al. 2016). The different types of electrochemical biosensor measurements have been reviewed by Stradiotto et al. (2003).

The most widely used potentiometric device is the pH electrode due to its simplicity, rapidity, low cost, applicability to a wide concentration range and particularly to its extremely high selectivity for hydrogen ions. Glass electrodes are composed of a thin ion-sensitive glass membrane and can monitor cations including sodium, lithium, ammonium and potassium (Stradiotto et al. 2003). Disadvantages to using pH electrodes are that they are bulky and invasive for tissue engineering applications, they require frequent recalibration, the glass tip can be easily damaged should always be kept wet to prevent dehydration of the hydrated glass gel layer on the external surface of the electrode.

The ion selective electrode is an example of an electrochemical biosensor and consists of an indicator electrode capable of selectively measuring specific ions. They are generally composed of a working electrode (potential is determined by its environment) and a reference electrode (potential fixed by a solution containing ion of interest at a constant activity) (Stradiotto et al. 2003). Since the potential of the reference electrode is constant, the value of the potential difference (cell potential) can be related to the concentration of the dissolved ion (Stradiotto et al. 2003). Amperometric biosensors have been widely used in point-of-care testing for applications such as monitoring glucose levels in people with diabetes (Hammond et al. 2016). Amperometric biosensors function by the production of a current when a potential is applied between two electrodes (Chaplin 2014). Some of the drawbacks of amperometric sensors are electrochemical interferences (detection of non-specific analytes), the lack or low response reproducibility, particularly since sensing biocomponents often have a limited lifetime. In addition, modifying the electrode surface to favour a single electrochemical process can be a difficult task. In addition, in the case of in vivo measurements biocompatibility and biofouling can be critical issues (Săndulescu et al. 2015). Conductometric sensors rely on changes of electric.conductivity of a film or a bulk material, whose conductivity is affected by the analyte present (Stradiotto et al. 2003). Thin films are used mostly as gas sensors, due to their conductivity changes following surface chemisorption (Stradiotto et al. 2003).

### Optical biosensors

**Fig. 6:**
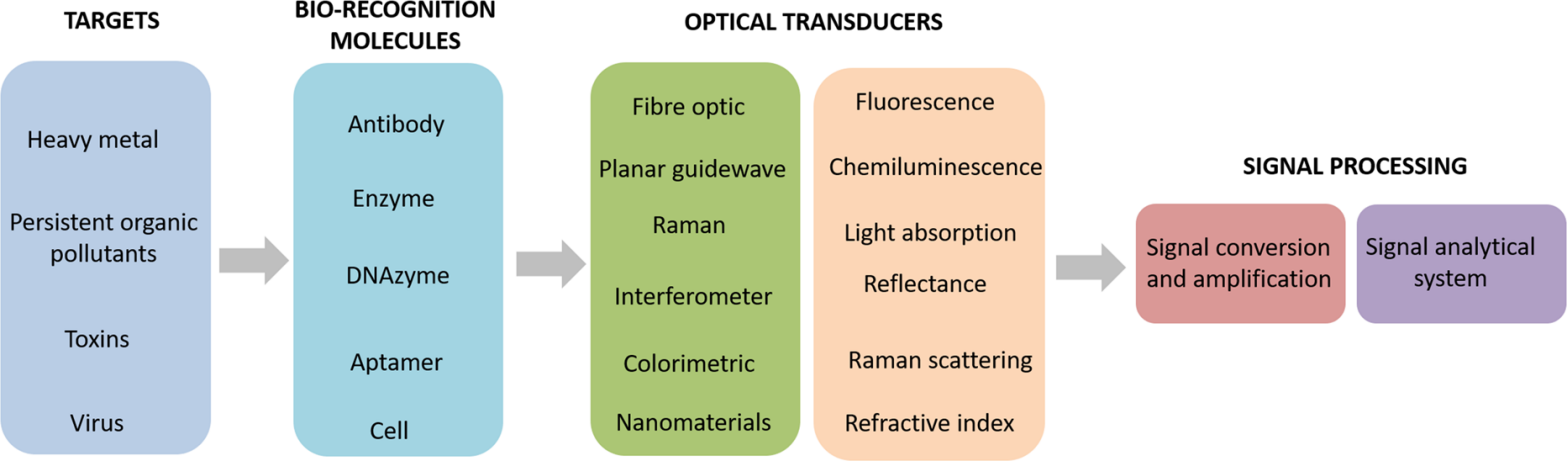
Optical biosensors are designed to target a molecule. Optical biosensors have biorecognition molecules specific to the target molecule, the signal is then optically transduced, and the signal is processed

Optical biosensors are one of the most common type of biosensor used for applications such as environmental monitoring, food safety, drug development, biomedical research, and diagnosis (Long et al. 2013). The main goal of optical biosensors is to produce a signal which is proportionate to the concentration of an analyte (Damborsky et al. 2016). Optical biosensors that exploit light absorption, fluorescence, luminescence, refractive index, Raman scattering and reflectance are powerful alternatives to conventional analytical techniques (Long et al. 2013) They allow rapid, highly sensitive, highly specific, real-time, cost effective detection of biological and chemical substances without any time-consuming sample concentration or prior sample pre-treatment steps. Figure 6 shows a schematic of a biosensor which displays the first stage as the target of interest, these are identified by biorecognition molecules, an optical transducer converts the signal into another signal form which can be amplified and analysed. Optical biosensors can be split into two main categories including label-free and label-based.

Label-free detection involves the generation of a signal directly by the interaction of the analysed material with the transducer. Whereas label-based involves the use of a label and the optical signal is then generated by a colorimetric, fluorescent or luminescent method. For example, glucose can be detected by enzymatic oxidation using label-assisted sensing. Jankowska et al. developed a biosensor based system to monitor pH and glucose concentration during wound healing (Jankowska et al. 2017). The hydrogel coating composed of a fluorescent pH indicator dye and a metabolite-sensing enzymatic system, based on glucose oxidase and horseradish peroxidase. Changes in metabolite and enzyme concentration in artificial wound extract were successfully converted into a fluorescent signal.

## Fluorescence probes for monitoring

Fluorescent probes can also be used to monitor the cellular microenvironment. This can be achieved by fluorescently labelling proteins of interest, delivering fluorescent nanoparticles, incorporating fluorescent protein tags and live cell dyes to investigate cellular processes under the microscope (Ettinger and Wittmann 2014).

### Fluorescent proteins

As mentioned, fluorescence monitoring can be performed in tissue engineering and regenerative medicine by fluorescently labelling proteins of interest. Some proteins or small molecules in cells are naturally fluorescent; which is known as intrinsic fluorescence or auto fluorescence and can be used to label live cells for monitoring (Jensen 2013). The chemically inert, green fluorescent protein (GFP) is an example common naturally occurring fluorescent protein sourced from jelly fish Aquorea Victoria (Tian et al. 1999). Upon excitation of UV or blue light, the GFP emits a bright green light. By creation of a genetic in-frame fusion of the fluorescent protein to a protein of interest, localisation of that protein to specific tissues, cells or subcellular compartments can be monitored and imaged non-invasively (Jensen 2013).

Fluorescent proteins can act as reporters by fusing the reporter gene to the promoter or coding sequence of a gene of interest, this will provide information on how much the gene or protein is expressed (Noguchi and Golden). Fluorescent proteins have many advantages and disadvantages as reviewed by Noguchi and Golden (Noguchi and Golden) and Jensen (2013). (1) They have a very bright fluorescent signal which is useful for visualisation of specific structures within cells (Noguchi and Golden). However, on the other hand the brightness emitted can be affected by temperature and can vary depending on the cell type (Jensen 2013). (2) The fluorescent proteins come in a variety of colours which can be fused to different proteins of interest within the same cell to study the co-localisation and expression of multiple proteins simultaneously (Noguchi and Golden). But care should be taken when selecting particular fluorescent proteins for cells, as for example Ds-Red fluorescent protein impairs the viability or growth of hematopoietic stem and progenitor cells (Jensen 2013). (3) Specific areas in a small area of tissue or cells can be excited by using confocal microscopy, which can also generate 2-D or 3-D images (Noguchi and Golden).

Disadvantages to using fluorescent proteins are: (1) Prolonged exposure to excitation light can generate free radicals (reactive oxygen species) which can damage DNA, RNA and proteins by oxidation, resulting in phototoxicity. (2) Moreover, it has also been shown that fluorescent proteins can induce apoptosis in cells, which indicates a possible reason for the difficulty in establishing stable cell lines expressing the protein. (3) Attaching a fluorescent protein to a protein of interest generally does not affect function, structure, and localization of a protein. However, in some cases, it can impair protein function and expression of this construct can adversely affect cellular function. (4) Prolonged exposure to excitation light causes photobleaching of fluorescent proteins which reduces their ability to fluoresce. (5) Cells contain compounds that exhibit auto fluorescence, therefore the signal from the fluorescent proteins need to be high enough compared to the auto fluorescence to rise above the background.

### Fluorescent nanosensors

Fluorescent nanosensors are sub-micron sized optical sensors specifically designed for non-invasive analyte monitoring in real time (Desai et al. 2014). They generally based on porous matrices composed of crosslinked polyacrylamide which encapsulate a sensing component that is responsive to analytes (Buck et al. 2004). such as hydrogen ions (Chauhan et al. 2011), calcium ions (Di Si et al. 2012), magnesium ions (Park et al. 2003), temperature (Chauhan et al. 2014), reactive oxygen species (Lavado et al. 2015), molecular oxygen (Chauhan et al. 2016; Giuntini et al. 2014) and glucose (Xu et al. 2002). Firstly, by encapsulating the sensing component such as an synthetic organic fluorophore, the matrix provides a protective coating which prevents interferences such as non-specific protein binding within a cell and protects the cell from potentially toxic effects of free fluorophores (Buck et al. 2004). Ratiometric fluorescent nanosensors have been developed which are composed of a fluorescent indicator dye, and a reference dye encapsulated within the matrix. The sensor response is based on the fluorescence emission intensity ration between the indicator dye and the unresponsive reference dye to the target analyte. By using a ratio a more accurate measurement of the analyte can be achieved (Buck et al. 2004). Since the production of fluorescent nanosensors, a number of ratiometric fluorescence nanosensors for pH (Chauhan et al. 2013; Orsi et al. 2015; Elsutohy et al. 2017) have been reported based on polymeric nanoparticles, silica nanoparticles, quantum dots, cellulose nanocrystals, latex nanobeads, and zeolite-based nanoparticles (Marín et al. 2012). Overall, fluorescent nanosensors are useful for sensing due to their small size, fast response, intense signal, against relatively low background noise, relatively simple instrumental set-up, and ability to monitor non-invasively (Harrison and Chauhan 2018).

### Quantum dots

Quantum dots (Qdots) are semi-conductor nanoparticles of a narrow size between (5–10 nm in diameter) and emit light if electricity or light is applied to them (Hasan et al. 2014). They are very photostable, with a long fluorescence life time and their fluorescence can be controlled by their size, for example larger dots may emit a red fluorescence, whereas smaller dots emit a green fluorescence. Quantum dots generally consist of a three layer-structure, composed of a core, shell and polymer coating. The most common quantum dots have a cadmium chalcogenide core which is usually coated with a zinc sulphide shell to improve photoluminescence. The outer surface of the quantum dot is usually modified so the dots can be directed to a target. The application of quantum dots is similar to the use of organic fluorophores and can be used for specific labelling of individual cell surface biomolecules. Jensen reviews the limitations of quantum dots. One of the major limitations is the toxicity of the quantum dots (Jensen 2013). This is due to the semiconductor material which are usually heavy metals embedded within the core, and the generation of free radicals during excitation. Since they are composed of heavy metals they are potentially toxic during in vitro imaging. Another issue is that quantum dots sometes have specialised coatings which make the overall molecule much larger than small organic dyes. This is more of an issue for cell internalisation and subsequent intracellular tracking. Since the fluorescence intensity of Qdots is highly stable and sensitive, fluorescence transduction based on chemical or physical interaction occurs on the surface either through direct photoluminescent activation or through quenching. Qdots have been widely investigated for possibilities of sensing pH, ions, organic compounds, and biomolecules (nucleic acids, protein, and enzymes), as well as other molecules of biological interest. While the toxic effects of some Qdots have still remained as a concern, the recent advancements in application of Qdots in tissue engineering to detect the enzyme and biomolecules are significant achievements of biosensing research (Hasan et al. 2014).

## Monitoring in bioreactors and microfluidics

As mentioned, a bioreactor is a vessel that allows biological/chemical reactions or processes to occur, which can be on an industrial scale. Bioreactors have been commonly used for applications such as fermentation for the production of ethanol (Roy et al. 2016), production of therapeutic proteins (Timm et al. 2015), viral vaccine production(Gallo–Ramírez et al. 2015). Being able to monitor parameters that affect biotechnological processes is important to ensure productivity and product quality (Reinecke et al. 2015). Parameters that should be monitored include temperature, pH, glucose, pO_2_, PCO_2_, and cell density within the culture medium (Reinecke et al. 2015). Bioreactor monitoring techniques can be placed in three main categories, including offline, inline and online (Lourenço et al. 2012). Offline measurements include manual or automatic sampling, transferring of a sample to a separate laboratory to be analysed, which often causes a delay in the analysis. Inline monitoring also includes manual or automatic sampling; however, the collected samples are analysed within close vicinity of the bioreactor. Online monitoring includes in situ measurement acquisition, where the sensing device is often incorporated into the bioreactor and the sample is typically not removed. The chemical components within bioreactor media are mainly monitored by offline methods that require a biomass separation step, such as high-performance liquid chromatography. However these methods can be time consuming and do not enable real time knowledge of the conditions affecting bioprocess performance (Lourenço et al. 2012). Tables 5 and 6 provides an overview of examples of where different kinds of transducers have been incorporated into biosensors.

Many microfluidic devices used for organ modelling have more recently began incorporating the ability to monitor the cellular environment. Being able to monitor the chemical environment can help improve understanding of cellular responses (Acosta et al. 2007). Oxygen is often a key component that is monitored within microfluidic devices. This is because oxygen is required for aerobic respiration and impacts cell viability, in addition oxygen tension can impact cell migration (Acosta et al. 2007). Being able to monitor oxygen levels in microfluidic devices is difficult, as conventional methods of oxygen sensing include the use of bulky probes. Compared with electrochemical methods, optical oxygen sensors also do not require a reference electrode and do not consume analytes which is crucial in micro-scale because of the low number of analytes available which can bias an accurate detection. Overall, it appears that optical chemical sensors are the most commonly used component for integration into microfluidic devices. This is because they are highly sensitive, inexpensive, easy to miniaturise and are allow non-invasive monitoring (Sun et al. 2015). Some of the demands of optical oxygen sensors include high brightness, capability to be applied as a thin film (below 1 µm thickness), good photostability, compatibility with sample, cheap or established imaging systems, simple and microfluidic production compatible preparation steps, compatibility with the chip materials and low or no toxicity (Sun et al. 2015). Shaegh et al. (2016) developed an optical multi-analyte sensing module integrated with a microfluidic bioreactor for in situ monitoring of pH and dissolved oxygen in the circulating culture medium. The real time pH monitoring was detected by the level of light absorption by the phenol red within the cell culture medium, and the oxygen sensing was achieved by measuring the degree of quenching in the luminescent intensity of an oxygen sensitive fluorophore. The advantage of this platform is that it is low cost and user friendly. It is also a miniature and compact detection system which is more desirable over bulky spectrophotometry or microscopy techniques (Shaegh et al. 2016). Being able to monitor the specific pH, it can indicate when circulating medium should be replaced with fresh medium. Whilst being able to monitor oxygen levels in bioreactors is important as changes in oxygen delivery to cells can cause variations in cellular metabolism and physiological pathways.

Shin et al. (2017) developed a human liver-on-a-chip microfluidic platform with integrated electrochemical biosensors, for the continual monitoring of the metabolic activity of the organoids by measuring the levels of secreted biomarkers for up to 7 days which agreed with the data acquired by ELISA. The versatile and robust microfluidic electrochemical biosensor was capable of automated and continual detection of soluble biomarkers, which is useful for long-term monitoring of human organoids during drug toxicity studies or efficacy assessments of in vitro platforms. The advantages of this system are the automation of the operation of the electrode, label-free antigen detection process requires minimum medium depletion; regenerative capability of the electrode surface upon saturation with captured antigens; and cost-effectiveness due to the use of the miniaturized electrodes and microfluidic platform, long term continual monitoring of biomarkers.

## Conclusion

In summary, tissue engineered scaffolds and bioreactors hold great potential for cell cultivation for future target biotherapeutics. Furthermore, they offer a range of additional advantages including (1) delivering nutrients and eliminating waste/metabolites, (2) mechanically stimulating cells, (3) building systemic models, (4) enhancing pathways for cell–cell signalling and co-culture, and (5) enables long term culture. Tissue engineered scaffolds mimicking extracellular matrix have also been fabricated to augment cell culture, by recreating innate microenvironments. Biosensors are the future of tissue engineered scaffold and bioreactors, as they enable non-invasive monitoring of the cellular microenvironment. Real-time monitoring of culture environments enables automatic realignment of ideal biochemical parameters, such as adjusting for nutrients and waste, to optimise cell growth. We anticipate that the encouraging developments in this field provide great promise to further under-stand the cellular microenvironment in tissue engi-neering and regenerative medicine applications.
